# Communicative and Methodological Challenges Related to Collecting Data with Older Adults with Dementia in Senior Living Communities During the COVID-19 Pandemic

**DOI:** 10.1177/00914150241300892

**Published:** 2024-12-08

**Authors:** Tamara D. Afifi, Charles E. Burnham, Nancy Collins, Chloe Gonzales, Aria Ma, Allison Mazur, Erin E. Naffziger, Kyle Rand, Yuval Rosen, Abdullah Salehuddin, Jennifer Stamps, Nikki Truscelli, Veronica Wilson

**Affiliations:** 1 Department of Communication, 8786 University of California Santa Barbara, Santa Barbara, CA, USA; 2 Rendever, 66-70 Union Square, Third Floor, Somerville, MA, USA; 3 Department of Psychological and Brain Sciences, 8786 University of California Santa Barbara, Santa Barbara, CA, USA; 4 Kinsey Institute, Bloomington, IN, USA; 5 Department of Communication and Information Sciences, Tuscaloosa, AL, USA; 6 Department of Communication, Central Oregon Community College, Bend, Oregon, USA

**Keywords:** virtual reality, zoom, ADRD, dementia, family members, COVID-19

## Abstract

The COVID-19 pandemic presented significant challenges to researchers collecting data with older adults, particularly older adults with Alzheimer's disease and related dementias (ADRD). The goal of this article is to articulate the communicative and methodological challenges and lessons learned from collecting data with older adults in senior living communities with mild cognitive impairment and ADRD and their adult children (who were geographically separated) during the pandemic. Communication was much more than what we were studying; it was essential to the success and ethical implementation of our research. We were working with a vulnerable population during a pandemic where recruitment, consent, and data collection required heightened and adapted communication strategies to reduce confusion, promote safety, and ensure data could be collected in an effective manner. The way we communicated with the participants, their networks, and the senior living communities was crucial to establishing strong human connections and subsequently successful data collection.

## Introduction

The COVID-19 pandemic presented significant challenges to researchers collecting data with older adults (see also Harwood, this issue; Sands et al., 2020; Sharma et al., 2022), particularly older adults with Alzheimer's disease and related dementias (ADRD). At the same time, it provided novel ways of thinking about technology like virtual reality (VR) and Zoom and how they could be used to improve the lives of older adults who are socially isolated. For many individuals, including older adults, learning how to use new technologies to stay connected to others during the pandemic-related lockdown became an essential part of everyday life (see also Otchere et al., this issue).

When the pandemic began in early 2020, we were collecting data in senior living communities for a Phase I Small Business Technology Transfer (STTR) grant through the National Institute on Aging (NIA; Afifi et al., 2021, 2023). The goal of that pilot study was to test the feasibility of using an emerging VR platform, Rendever, with older adults with mild cognitive impairment (MCI) or mild to moderate ADRD and their family members who lived at a distance. We gathered data from 21 older adult-family member dyads in one senior living community before having to suspend data collection in a second community due to the COVID-19 shutdown (see also Cooper this issue; Otchere et al. this issue). This pilot study tested the efficacy of the remote features of the Rendever platform with those dyads. It included a baseline telephone call between the older adult and their family member who were geographically separated, followed by use of the VR once a week for three weeks, with surveys and interviews immediately following each VR session.

The Rendever VR platform uses networking technology and a live communication component that allows multiple people to experience the same VR content simultaneously and communicate with each other while doing so, even if they are in other parts of the world. An adult child and older adult could put on the synced headsets and see and experience the same virtual experiences in the headset simultaneously while talking to each other (similar to a speaker phone inside the headset). They could swim with the dolphins or travel anywhere in the world together, co-view and reminisce about the house they grew up in or where they went to school using Google StreetView, and co-view family photos and videos inside the VR. Our research team ran the VR for the older adults in the senior living community and the family members were shipped a headset and trained how to set it up and use it. For older adults with cognitive and physical challenges, this technological ability helps them continue to thrive both relationally and personally (Afifi et al., 2021, 2023).

The results from the Phase I study showed that the older adults with MCI and ADRD and family members found the VR safe, extremely enjoyable, and easy to use (see Afifi et al., 2021). Human and automated coding revealed that older adults were more conversationally and behaviorally engaged with their family member in the VR compared to the baseline telephone call and in the VR sessions that used reminiscence therapy (where they co-viewed family photos and videos and visited personally meaningful addresses from the past, such as one's old house, rather than traditional virtual adventures). The VR was also associated with improvements in older adults’ affect and stress, relationship satisfaction, and overall quality of life, compared to baseline (see Afifi et al., 2023). Family members’ negative affect, depressive symptoms, and caregiver burden also decreased, and their mental health improved after using the VR, compared to baseline. In addition, older adults and family members who experienced the VR sessions as more socially engaging reported better psychological and relational well-being, with older adults also experiencing improvements in overall quality of life.

We then conducted a larger Phase II STTR clinical trial (i.e., the Thrive Study) from NIA where we collected data from 186 older adults from 23 senior living communities and one of their adult children who lived at a distance in 2021–2024 during the pandemic. In this clinical trial, the older adults (half with MCI and half with mild to moderate ADRD) were randomly assigned to use VR or Zoom with an adult child once a week for four weeks. Most adult children lived in other parts of the country or world (from eight countries). Surveys were collected at baseline, immediately after each technology session, after the intervention, and at 1 and 3 months post-intervention. We also conducted interviews throughout the study and the technology sessions were operated in-person by our research teams in the various senior living communities. The adult children were shipped VR headsets or set up on Zoom and trained how to use the technology before the study began. Surveys and interviews were completed in-person by the older adults and online by the adult children.

Both grants involved a partnership between the research team at the University of California, Santa Barbara (West Coast) and the research team at Rendever in Boston (East Coast). We actively collected data from senior living communities on both coasts at the same time. Despite the challenges associated with collecting data during the COVID-19 pandemic, both studies were extremely successful. In the Thrive Study, we recruited 186 dyads with no significant side effects or adverse events caused by the study or the technology, minimum disruption to the data collection itself, and a low attrition rate of 11% for the intervention and 15% for the follow-ups. The goal of this article is to articulate the communication challenges and lessons we learned from collecting the data from the Thrive Study. Some of these lessons were learned because of the pandemic, whereas others were more typical challenges associated with working with a community-based sample of this nature. Communication was instrumental to the success and ethical implementation of our research. Everything from recruitment, consent, and data collection required heightened and adapted communication strategies to reduce confusion, promote safety, and ensure the data could be collected in an effective manner. The older adult participants in our samples were also embedded within larger family and senior living community systems, making the coordination of communication among these networks a crucial component. Most importantly, the way we communicated with the participants and their networks was crucial to establishing and maintaining strong human connections and subsequently successful data collection.

## Consent, Recruitment and Enrollment Challenges and Strategies

### Consent Challenges and Strategies

One of the challenges of collecting data with older adults with ADRD is how to communicate and gather consent, which some of them might not be able to provide. Working with individuals with ADRD, particularly when considering consent, made us much more aware of how we communicated. Because of the risk and ethics involved, it is important that researchers have a specific, detailed plan about how to assess whether a participant can provide consent and how to determine changes in their communicative ability to provide consent throughout a study. Because this study also involved the adult child of the older adult in a senior living community and potentially a legal representative if the older adult had ADRD (if it was not the participating child), we had to secure multiple levels of consent for each dyad to participate in the study.

Our consent process followed numerous steps. Before the study began, the Director of each community emailed or mailed a letter to all of their older adults and their family members announcing the study and its purpose. We also asked the adult children to let us know if they did not want our research team reaching out to their parent. The letter to the families was particularly important because it enhanced the study's credibility and reassured the family members who were often very protective of their loved one's privacy. During this process, the research team formed strong partnerships with the executive directors, activity directors and other staff in the senior living communities, who worked with us to create a list of all eligible older adults who qualified for the study and screened them for a history of epilepsy, current hallucinations, vertigo, and paranoia, among other factors.

Potentially eligible residents were invited to talk with the researchers in-person to determine if they were interested and eligible to participate. The researchers explained the purpose and procedures of the study and gathered initial verbal consent to participate. At this time, researchers administered the Mini-Mental State Examination, Version 2 (MMSE-2) to verify eligibility. If an older adult's ability to consent was uncertain, the researcher completed a human subjects board-approved screener assessing their decision-making capacity to consent, and consent was obtained from the older adult's legal guardian. The legal guardian or adult child also completed the 8-item Informant Interview to differentiate aging and dementia measure (AD8) to help verify their parent's dementia status. This additional information was valuable because seniors can become accustomed to cognitive tests and highly educated individuals can score higher on the MMSE which can mask their true level of ADRD (see Borson et al., 2013).

How consent was gathered and communicated to the participants was essential to both projects, given that half of our older adult participants had dementia. In addition to providing verbal consent, the older adult, adult child, and legal representative all provided written consent. A simplified consent script was read aloud to residents with mild to moderate ADRD to ensure they understood the study procedures and associated risks and safeguards. The explanations of the study and onboarding process with the adult children and legal representatives typically occurred through Zoom. Importantly, the researchers also re-consented older adults with mild to moderate ADRD before the beginning of every technology session using a verbal consent “checklist” whereby the researcher stated the purpose of the study, what would happen, the risks, and the benefits in very plain language and asked the older adult if they understood each question. If the older adult was unable to answer the questions and/or was not alert and oriented to their surroundings, or if the older adult reported that they did not want to participate, then the session did not proceed that day.

One of the greatest lessons learned during the Thrive Study was to create as much structure as possible when working with older adults with ADRD and their families, but to be positive in the way we communicated and adapt easily to unexpected circumstances. The research team gathered information from the family members and staff that worked closely with the older adults with ADRD to determine particular triggers and times of day, word choices, and/or people that helped calm them. This not only helped the consent process, but also reduced the study's attrition rate. For instance, if the older adult had a spouse or other care provider living with them, we asked the spouse/care provider to walk their partner to the room where we were collecting data. The nonverbal and verbal communication of the spouse/care provider created calming and reassuring signals. They also sometimes helped explain what they would be doing that day in a way their loved one could receive. Finally, even though we restricted the days and times available to the adult children to engage in the VR sessions with their parent, working with families meant adapting to their busy work and parenting schedules. In essence, creating structure was essential to the data collection but communicating a sense of humanity and “meeting the person” where they were at with their cognitive state or family situation was more important.

### Recruitment Challenges and Strategies

The most challenging aspect of the Thrive Study was the recruitment of the older adults and their adult children. The recruitment of dyads for our Phase I pilot study was incredibly smooth and fast because we collected data from only one senior living community, and it was right before the pandemic. Based upon that pilot study, we expected that we could collect enough participants to secure our goal of 186 dyads from 12 senior living communities in a year and a half for the Thrive Study. Instead, we needed twice as many senior living communities and an extra year to reach that goal. We screened 1163 older adults to enroll 186 dyads.

There were two primary reasons why our expectations were unrealistic. The first is that the senior living community from which we collected our Phase I study pilot data was a partner with whom we had built a long-standing, mutually beneficial relationship for five years. The Director and staff trusted us and knew us extremely well. As a result, they allowed us to talk openly with the older adults about the study and actively encouraged them and their family members to participate in the project. This was an ideal community-engaged partnership. In Phase II, we did not have the opportunity to develop those same relationships with many of the other communities because of the intense recruitment schedule coupled with pandemic-related restrictions.

The second reason our timeline became unrealistic was the timing of the pandemic itself. The Phase I pilot data were collected immediately prior to the COVID-19 pandemic, whereas the Thrive Study was conducted during the middle and end of the pandemic. During the pandemic, the staff in the senior living communities was experiencing significant exhaustion and burnout from lockdowns, vaccinations, their residents contracting COVID-19, residents passing away, stress from family members, and staffing shortages. The family members were difficult to recruit because they were also exhausted from the long-term demands of the pandemic. In addition, wearing face coverings made recruitment arduous because the older adults who were hard of hearing could not read our lips. This was particularly problematic for older adults with ADRD, given that facial expressions often communicate warmth and trust and can reduce uncertainty and anxiety (Ellis & Astell, 2017). Some East Coast residents also expressed mistrust of the study due to its funding by the National Institutes of Health (NIH) (see also Harwood, this issue), which was the target of anti-vaccination rhetoric during the pandemic. Finally, we experienced a number of delays in starting data collection and recruitment due to COVID-19 outbreaks, tested daily for COVID-19 before entering a senior living community, and increased and continuously evolved our COVID-19 safety protocols (including using Cleanboxes which sanitized the VR headsets with UVC light immediately after use).

Another challenge, regardless of the pandemic, was recruiting an ethnically and socio-economically diverse sample of older adults. Researchers have made remarkable breakthroughs in improving the quality of life of older adults in senior living communities through technologies like VR (e.g., Afifi et al., 2023; Li et al., 2021). However, the primary beneficiaries of these technologies have been older white adults who can afford the senior living communities where they have been implemented. For instance, Hispanic older adults are more likely to live at home and be cared for by their family members, and when they do need long-term care, they are more likely to reside in nursing homes that are poorly run, understaffed, and deficient in performance (Cardenas et al., 2023). In both of our studies, we wanted to test the VR technology with a large number of older adults who were gathered in pre-existing locations in a safe environment, which happened to be senior living communities. Unfortunately, most senior living communities lack ethnic and economic diversity.

Numerous communication approaches were used in the Thrive Study to address some of these recruitment challenges. We relied on key staff at the senior living communities to communicate with the older adults who qualified and their adult children, informing them about whom we were and the importance of the study. The relationships we built with the staff helped us connect emotionally with the participants. We held town halls with the older adults and Zoom information sessions with their adult children. Additionally, we met with older adults in small groups to explain the study and build enthusiasm, followed by private meetings to screen them and assess their interest. To further build relationships, we spent time playing musical instruments for the older adults and organized ice cream socials once COVID-19 restrictions were lifted. After a family was enrolled, snowball sampling proved effective, as the older adults often discussed the study with their friends and encouraged them to participate. The entire recruitment was grounded in communication and fostering human connections through gatekeepers and time spent getting to know one another.

To increase the ethnic and socio-economic diversity of our sample, we recruited from five low-income senior living communities, including one primarily Spanish-speaking housing community. We hired and trained several Spanish-speaking research assistants (RAs) to translate our study materials and run study sessions with participants whose first language was Spanish. The residents in lower income housing likely benefited more from the technology than residents who had greater resources and ability to purchase new technologies and travel. The older adults in the low-income senior living communities also had greater mental health challenges, substance dependence, and accessibility challenges than older adults in the other communities. We adopted a person-centered approach in our communication to determine what the individual participant needed to be successful in the study. That included gathering a verbal history of likes and dislikes, checking in with the Directors of the senior living communities to ensure the safety of their residents, providing information on resources outside the community when needed, or sending a Wi-Fi hotspot to the adult child so they could have a stronger internet connection.

### Constructing and Administering Surveys

Another challenge numerous researchers confront is how to collect quantitative data when individuals have ADRD (see Perfect et al., 2021). In addition to using pre-established measures that were already shown to be reliable with older adults with dementia, establishing the reliabilities for many of the measures in our pilot study helped us determine what types of data collection methods and measures were going to work most effectively for the Thrive Study. Communication once again became central to our data collection. Using very simple language when we verbally explained each survey question, visual images of items, and pointing to items as we went through the surveys were incredibly helpful tools.

The surveys also went through multiple iterations after feedback from participants. Early in the Thrive study we found that the length of the surveys created frustration and was a barrier to completion for some participants. We reduced fatigue by reducing the number of scales and items, particularly if items were repetitive.

In addition to adapting the survey instrument, we adjusted the presentation of the survey for the older adults. Our older adult participants were managing a myriad of health issues and disabilities, including mild to moderate ADRD, poor eyesight, limited physical mobility, hearing loss, arthritis, hand tremors, and varying communication skills and abilities. Therefore, our team found it useful to have one lead researcher and assistant consistently assigned to each older adult participant. The researcher could then build trust and rapport with the participant to ensure their safety and comfort throughout the study and understand if any accommodations were needed for the older adult with dementia to feel comfortable participating and answering our questions (Novek & Wilkinson, 2019). As communication accommodation theory would attest (see Giles, this Issue), having the same researcher every session allowed them to better adapt their communication patterns to those of the older adult.

Our research team also adapted how we administered the surveys to the older adults. Older adults vary in their comfort with and trust in technology (Hope et al., 2014; Mitzner et al., 2010). Consequently, our research team used paper surveys for the older adults with MCI and sent online survey links to the adult children who lived at a distance. We also found that it was imperative to give participants autonomy to make decisions for themselves, and we accommodated their decisions to the best of our ability (Dewing, 2008; Novek & Wilkinson, 2019). If the older adult had MCI, they could either read the survey themselves and complete it with some assistance, or let us know if they would prefer a researcher to read it aloud to them. If the older adult had ADRD, poor eyesight, difficulty processing information, hand tremors, and/or significant arthritis, the survey was administered to them verbally.

If a researcher was reading the survey aloud to the participant, the participant may not have been able to retain the questions and answer options. To aid in this method of survey delivery, our research team put the anchors for the scales in a PowerPoint presentation on a laptop that was shown to participants alongside the survey. We could adjust the text size, so that questions and answers were legible. The PowerPoint was especially beneficial for participants who were hard of hearing (particularly given our face coverings) or had difficulty processing information. Lastly, displaying the answer choices on the slides helped those with processing issues by allowing them to easily reference and reread the options.

For older adults with dementia, we also used a “decision tree” to communicate the responses to the closed-ended Likert-type questions (e.g., strongly disagree to strongly agree). The researcher would start by asking the participant if they simply agreed or disagreed with the statement. Then the participant was asked if they (dis)agreed a little or a lot with the statement. This “decision tree” technique allowed participants to only choose between two options, rather than several, at a time, which aided in information processing.

## Technology Challenges and Opportunities

### Internet Connection and Hardware Challenges

Both the VR platform and the video conferencing tool used in the Thrive Study required internet access to operate the study activities. One technological challenge experienced in both studies was having a steady connection to Wi-Fi at the participating senior living communities. While the vast majority (>90%) of communities had Wi-Fi, the connection was sometimes unstable, resulting in lagging, distorted audio, and dropped connection. Unstable Wi-Fi connection was explained in part by the layout of the senior living communities, restrictions in the internet access, and the adult children's Wi-Fi. We were able to mitigate these issues by using hotspots and connecting with the senior living communities’ IT departments. Future studies should carefully consider the availability of Wi-Fi in their demographics, budget for hotspots, and work closely with IT teams to identify potential barriers prior to beginning data collection.

One issue with the hardware was audio fidelity. Participants in the VR condition occasionally reported having difficulty hearing and understanding their dyad partner through the headset. This is likely due to a number of factors, including internet connectivity issues, the quality of the headset microphone, and preexisting hearing loss (Lin et al., 2011). Microphone quality is difficult to improve upon without a cost-prohibitive upgrade in headset hardware. Perhaps a small number of more expensive headsets with higher quality microphones could be employed for situations where audio issues become problematic. When restarting the equipment and establishing a stronger internet connection did not work, a last resort solution in the Thrive study was to simply establish a telephone call between both participants using their cell phones on speaker in place of the native headset audio. This solution was applied at least once, and it allowed the technology sessions to progress much more smoothly once full communication was established.

### Accessible Technology

Even though the use of Zoom became more common during the pandemic, it is important to consider the wide range of technology experience in prospective participants (Pratama et al., 2020). Research suggests that while older adults may be initially uncomfortable with VR, they quickly acclimate to the technology and are able to use it safely and comfortably after some initial exposure (Afifi et al., 2021; Huygelier et al., 2019; Li et al., 2021). Thus, spending time to train participants on VR/Zoom was a key approach used by the study team. This training took place during the onboarding phase of both studies and allowed the study team to troubleshoot internet connectivity issues, address any concerns, improve comfort and familiarity with the technology, and foster a sense of connection with study participants. In addition, the adult children participating remotely received a Welcome Guide that included key information on Zoom or VR, basic troubleshooting instructions, a study calendar, and contact information for instant technology support.

Another key component that led to the success of this study was the intentional design of the VR platform allowing participants to focus on the experience. Rendever was created for older adults with accessibility and ease of use at the forefront of the design. The VR headsets are wireless, lightweight, portable, and easily adjustable. The study sessions at the senior living communities took place in a private room and in the same location each week. If a participant had social anxiety or physical limitations that prevented them from coming to the research room, however, the portable equipment allowed us to conduct the study in their room. The virtual visits were facilitated by a researcher using an Android tablet, removing the need for study participants to guide the experiences themselves or manually adjust the volume, which allowed them to fully engage in the virtual visit. Avatars were also used in one of the sessions when viewing family photos and videos in the VR, and when participants were waiting for their VR session to begin. Avatars have been shown to be a successful tool to increase embodiment and immersion (Waltemate et al., 2018). Older adults worked with the researchers to customize the avatars, creating one that represented themselves and their adult child (e.g., skin, hair, eye color). As we found out later in the interviews, some of the older adults were confused about the purpose of the avatars or that they were supposed to represent themselves.

When working with older adults with ADRD, it can sometimes be difficult to know when they need extra help or assistance. During the initial visit the researcher walked through what to expect during the first virtual visit and gave an overview of the VR technology. Each participant was carefully instructed to let the researchers know if they started feeling uncomfortable, dizzy, or wanted to stop the study. In addition, they were told that they could take the VR headset off or ask for it to be removed by a researcher at any time. Even with this training, some older adults with ADRD may become scared or uncomfortable when using technology, but will not always voice their concerns unprompted. For example, in one instance, a pop-up window appeared on the laptop screen during a Zoom session and the participant did not know how to close it. The participant did not immediately raise this concern to researchers. Though disruption from researchers should be kept to a minimum, periodic check-ins with participants can be helpful. In our experience, one check-in during the middle of the 20-min Zoom session was unobtrusive and effective at making sure participants did not encounter any difficulties. In a few instances, older participants in the VR group reported experiencing mild nausea toward the end of the session, but they did not disclose this until the survey portion of the session. To ensure participants remain safe and comfortable, it is important to establish rapport and clear expectations. Participants should feel comfortable asking for water, a break, or help with the technology. Older adults with ADRD sometimes experience anxiety when interacting with technology that is unfamiliar to them, so it is important to reassure them that the researchers can easily assist them or operate the equipment for them (Kim et al., 2023).

The participants in this study also had varying levels of engagement with technology in their day-to-day lives. Some were regular users of Zoom or FaceTime, while others struggled to keep up with their landline telephone. As life has become increasingly digitized, barriers to independence rapidly emerge to those left behind; access to modern technology can be partially bridged by education, encouraging independence in older adults, but physical and cognitive health realities of aging often present challenges to those who try and adapt to modern technologies (Pirhonen et al., 2020). The economic pressure of aging, especially in lower-income older adults, further impacts technological literacy (Li et al., 2021).

Particularly since the COVID-19 pandemic, there has been an increase in older adults’ use of technology, which is a good reminder that technology should be designed with this growing demographic in mind (see also Drazich et al., 2023; Cooper, this Issue). One lesson that participants communicated to us was the benefit of having a technology facilitator, which we provided in both the VR and Zoom conditions. Rendever's platform was designed to have a facilitator operating the system, but this extends to Zoom as well. Both Zoom and VR also presented unique benefits over a standard phone call for people with advancing cognitive decline, including the ability to read lips and see their family member's surroundings and other nonverbal communication in Zoom.

Overall, when thoughtfully delivered and intentionally tailored to specific user needs, including older adults living with MCI or ADRD, a new technology can become a welcomed surprise instead of a dreaded frustration. In each of the participating communities, we often encountered skepticism, with many older adults feeling they were beyond the need to learn new technology. However, our continued friendliness, their curiosity, and encouragement from family members helped these older adults overcome their initial fears and reservations. We learned during recruitment to emphasize that the study was designed to foster important family connections, facilitated by the new technology, rather than emphasizing that the study was about the technology itself. We also assured participants that the research team would handle the technological aspects of the study, and that their only responsibility was to attend the study sessions.

## Working with Families and Community Partners

### Logistical Considerations in Family-Centric Research

Our Thrive study design required a comprehensive strategy that balanced the need for detailed planning with the flexibility required to accommodate the unpredictable aspects of working with older adults with ADRD and the busy schedules of adult children who were balancing careers, children, and long-distance caregiving. Because we were sometimes collecting data in multiple senior living communities at once on both the West and East Coasts, it worked best to recruit as many families as possible until a full cohort was ready to begin the study. The project manager would then choose days and times that the research team would be in a particular community, and provide those time slots to the adult children. Times were carefully chosen to avoid overlap with community activities and to avoid times of day that were less optimal for participants (such as late afternoons or early evening hours when individuals with ADRD might be experiencing sundowning: Canevelli et al., 2016). Fortunately, most adult children were able to use the technology from their home or office; we then coordinated their availability with that of their parent. Even with these coordinating efforts, we often had to adapt and reschedule sessions when unexpected issues arose (e.g., an older adult fell or was too disoriented to participate that day, or an unexpected work conflict arose).

An essential element of our approach was implementing a participant-centric scheduling protocol. This innovative system combined the use of digital scheduling tools (e.g., Google calendar to organize our research team and some adult children), which provided real-time updates and adjustments, with traditional communication methods, such as visits with the older adults, phone calls and email reminders, to ensure inclusivity and accessibility for all participants. Recognizing the importance of minimizing disruptions to the daily lives of older adults, we worked closely with senior living community staff to integrate study sessions into the existing schedules of community activities and personal routines. This collaboration ensured that our research activities harmonized with participants’ daily lives, enhancing participation rates without compromising their regular engagements. In addition to having the same researchers meet with the same older adult each week, we created visually-based scheduling sheets for each older adult at the onset of the study, which included photos and names of the research team members with whom they would be working. This planning and consistency were instrumental in building trust between participants and the research team, which, in turn, facilitated smoother interactions, enhanced the older adults’ recognition and rapport with the researchers, and fostered their investment and engagement in the technology sessions (see also Phinney et al., 2007).

### Interpersonal Dynamics in a Sensitive Research Context

When conducting research with individuals with cognitive decline and their families, an understanding of interpersonal communication is paramount. Our methodology was rooted in an emphasis on empathy, respect, and person-first language (Alsawy et al., 2020; Kitwood, 1997). For example, the words “dementia” or “Alzheimer's” were not used in the recruitment material or conversations with participants in order to avoid potentially making the older adult or adult child feel stigmatized (Jellinger, 2010; Rewerska-Juśko & Rejdak, 2020; Werner et al., 2012). The research team avoided language that would suggest the presence of ADRD since emotional reactions to dementia diagnoses can be deeply affecting (Aminzadeh et al., 2007). This approach was critical in navigating the complexities of engaging with a vulnerable population and fostering inclusive and respectful relationships with the participants (Nguyen & Li, 2020; see also Giles, in press).

Training for the research team was rigorous, prioritizing the cultivation of an environment where the personhood of individuals with cognitive decline was at the forefront of every interaction. Before the study began, the project manager spoke at length with the adult child and gathered an oral history of their family and the older adult. The project manager also interviewed the older adult in-person and gathered information about their hobbies, interests, family relationships, and past. This allowed us to personalize the older adults’ VR sessions and coach the adult children on how to engage with their loved ones during the reminiscence therapy VR sessions. Even though most reminiscence therapy evokes positive affect in older adults with ADRD, it can also evoke negative affect if it reminds them of painful memories from their past or longing for the past (Henkel et al., 2017; Lazar et al., 2014). This training allowed us to guide adult children on how to communicate with their parents and draw upon stories that were positive and meaningful, and how to avoid questions, memories, people, and language that could be frustrating or triggering if their parent had ADRD or depression. The training for our research team also underscored the importance of language and terminology that recognized the dignity and humanity of participants such as verbalizing understanding, giving ample time for responses, and expressing positive nonverbal cues, such as nodding and smiling (see also Kim et al., 2024). The researchers avoided linguistic choices that could inadvertently cause distress or offense such as ignoring, talking over, quizzing, and correcting the older adult (Williams et al., 2023). In addition, elderspeak (i.e., infantilizing language that derives from stereotypes of older adults as incompetent) was avoided (Giles et al., 2022; Shaw & Gordon, 2021; Williams et al., 2009; see also Fowler et al., 2015). This language represented our commitment to treating participants with the utmost respect and consideration.

Our interactions with participants were informed by more than just the requirements of data collection; empathy and compassion were integral, translating into practical actions. For instance, engaging with participants often meant allocating considerable time to actively listen to their stories and concerns, extending beyond the confines of the study's immediate objectives (Edvardsson et al., 2008; Fazio et al., 2018). Adjusting communication strategies to align with participants’ cognitive abilities was a key premise of our approach. This often involved being sensitive to the verbal and nonverbal behaviors of the older adults and making necessary accommodations (see Giles et al., 2023; Staehler et al., 2022), demonstrating our adaptability and commitment to making the study accessible to all participants. This method used communication skills such as asking short questions in the present tense, picking up on emotional cues, not rushing the person with cognitive decline in their responses, not dominating the conversation, and letting the person with cognitive decline have agency in steering the conversation (see also McEvoy & Plant, 2014). This approach often meant that we went over the allotted time originally devoted to each study session.

Moreover, understanding and communicatively navigating family dynamics was crucial, especially given the challenges posed by cognitive decline (see also Faw & Johnson, this issue; Lin & Silva, this issue). For instance, Tatangelo et al. (2018) found that different knowledge of ADRD among the family members, beliefs about caregiving, expectations for the caregiving role, and general lack of communication surrounding the care of the individual with ADRD were significant stressors for adult child caregivers. Thus, the embedded relationships and belief systems curated through the family's socializing and history together served to provide the necessary context to interpret caregiving stress.

Research also shows that past familial experiences can have a lasting impact on various domains over one's life course, including well-being and social/interpersonal functioning (Paradis et al., 2011). In our Thrive study, two types of family history experiences, *unresolved hurt feelings* between family members (Miller & Roloff, 2014), and *parental (dis)favoritism* (Finzi-Dottan & Cohen, 2010) interfered with our recruitment and data collection the most. First, *unresolved hurt* is defined by involuntarily ruminating about hurtful events from the past. Family histories consisting of unresolved hurt feelings created challenges during data collection. For example, one older adult with ADRD jokingly referred to his adult son's VR avatar as a “dummy” because it looked like a cartoon character. However, this remark distressed his adult son, bringing up old feelings of hurt in their relationship. After several sessions where this surfaced, it prompted a conversation with the researcher about those feelings. Second, *parental (dis)favoritism* refers to children's perceptions of differential treatment by parents that is consistently one-sided (i.e., praising, supporting, and/or rewarding one child more than others; Jenkins et al., 2003). The negative effects of parental (dis)favoritism manifested as disruptions in the study during the recruitment phase. In several instances, the legal representative of the older adult – who was not the intended child participant in the study – refused to allow their sibling to participate alongside their parent due to unresolved feelings of hurt. In some cases, they believed their parent favored their sibling or felt their sibling had not contributed enough to caregiving duties (because they lived out of town) to “earn the right” to be included in the study. It is crucial to anticipate these issues and have a clear study plan for responding to them when they arise.

Taking family history into account (e.g., Bernhold, in press), the potential for VR experiences to evoke strong emotional reactions was a critical area of focus. Our team was prepared to navigate the gamut of responses, from the joy of revisiting cherished memories to the distress that might arise from immersive simulations of past experiences. Recognizing and validating these emotional responses are essential (Hummert, 2023), as is the capacity to offer immediate emotional support or referrals to professional services if the engagement unearths deeper psychological distress.

### Collaborative Engagement with Senior Living Communities

Our collaboration with senior living communities emerged as a cornerstone of our study's success, embodying a partnership that was both instrumental and reciprocal. This communicative collaboration was initiated through early engagement activities aimed at comprehensively understanding the specific needs and preferences of the senior living communities involved. A commitment to transparency and mutual benefit guided this partnership, ensuring that our research objectives not only aligned with, but actively supported, the interests and well-being of the communities.

Central to our engagement strategy was the principle of giving back to the communities, a commitment that extended beyond the dissemination of our findings. We sought to contribute meaningful resources for mental health support and to organize educational events designed to enhance the communities’ understanding of technology and its potential benefits for older adults. After data collection for the Thrive Study concluded, we went back into several of the senior living communities and had VR weekends with the older adults, staff, and undergraduate student volunteers. We also created service-learning opportunities in a few of our university classes, and took the students to one of the participating low-income senior living communities to provide free workshops on conflict management (which the Director of that community deemed their highest priority). The allocation of additional funding received through research awards was also put toward mental health initiatives within the low-income senior living communities. Many of our faculty, graduate students, and undergraduate RAs now have long-standing relationships with the staff and older adults in the senior living communities.

Communicating regular updates to the communities was another vital component of our engagement strategy, involving organized meetings and forums where researchers shared ongoing progress of the Thrive Study. This openness not only fostered trust but also provided opportunities for the community to actively contribute to the research process. Valuable feedback collected during these sessions allowed us to refine our study protocol and VR content in response to community needs, thereby enhancing the relevance and impact of our research. Once results of the clinical trial (currently being analyzed) are ready for dissemination, a newsletter with the findings will be distributed to all participants. Town hall meetings will also be held in all participating senior living communities, and in other communities that are interested in learning about the findings.

Our study's community-engaged research approach underscored the importance of cultivating strong, reciprocal relationships with senior living communities and stakeholders. By prioritizing early engagement, transparency, mutual benefit, and a commitment to giving back, we conducted a study that successfully met its research objectives while delivering meaningful benefits to the communities involved.

## Conclusions

Although collecting dyadic, longitudinal data with older adults with ADRD and their adult children presented numerous challenges, especially during the COVID-19 pandemic, the study design and technology employed helped minimize scheduling barriers by removing the necessity for participants to physically commute to the senior living communities. At the same time, future research should consider communicatively engaging with the substantial portion of older adults who age in place at home. Leveraging the flexible networking capabilities of the VR platform enables individuals to share experiences with others in separate geographic locations. Introducing this accessible technology into home settings will be a powerful tool to improve relationships, reduce social isolation and loneliness, enhance cultural/ethnic and socio-economic diversity, and improve feelings of well-being. Due to the user-friendly design of the VR interface, individuals with visual impairments or limited technology experience can still engage in virtual activities by enlisting the assistance of a trusted loved one, caregiver, home-based healthcare service, or volunteer. In fact, one unexpected finding in our research was that numerous older adults who were legally blind could see images much more clearly when using VR than without it.

The lessons learned from our research underscore the importance of communication in the research process and the practice itself. Communication is always important when we are conducting research. When working with vulnerable and complex populations like older adults with ADRD and their family members, however, our communication becomes more mindful and consequential. Integrating innovative technologies like VR in sensitive and dynamic environments also requires a careful balance between logistical precision, interpersonal sensitivity, and ethical considerations. Future research should build on these findings, exploring not only the efficacy of such technologies in enhancing the quality of life among older adults but also the broader implications for family communication, community engagement, and societal perceptions of aging and technology (see also Wei et al., 2023). As we continue to push the boundaries of what is possible in enhancing human health and well-being through technology, the lessons from this study serve as a foundational guide for navigating the challenges and opportunities that lie ahead.
